# Polyimide aerogels with novel bimodal micro and nano porous structure assembly for airborne nano filtering applications

**DOI:** 10.1039/d0ra03907a

**Published:** 2020-06-16

**Authors:** Shahriar Ghaffari Mosanenzadeh, Zia Saadatnia, Solmaz Karamikamkar, Chul B. Park, Hani E. Naguib

**Affiliations:** Department of Mechanical and Industrial Engineering, University of Toronto Toronto Ontario M5S 3G8 Canada naguib@mie.utoronto.ca; Department of Materials Science and Engineering, University of Toronto Toronto Ontario M5S 3G8 Canada

## Abstract

Aerogels have presented a very high potential to be utilized as airborne nanoparticles' filtration media due to their nanoscale pore size and extremely high porosity. The filtering performance of aerogels, such as air permeability and filtration efficiency, is highly related to the configuration of aerogels' nanostructure assembly. However, as aerogel morphology is formed with respect to the intermolecular forces during the gelation stage, tailoring the aerogel nanostructure assembly is still a challenge. In this work, a novel strategy for tailoring polyimide aerogel nanostructure assembly is proposed by controlled disturbing of the intermolecular forces. From the results, the nanostructure assembly of the 4,4′-oxydianiline (ODA)–biphenyl-tetracarboxylic acid dianhydride (BPDA) polyimide aerogel is tailored to a uniform bimodal micro and nano porous structure. This was achieved by introducing the proper fraction of thermoplastic polyurethane (TPU) chains to the polyimide chains in the solution state and through a controlled process. The fabricated polyimide/TPU aerogels with bimodal morphology presented enhanced filtration performance, with 30% improved air permeability and reduced cell size of 3.51 nm over the conventional ODA–BPDA polyimide aerogels. Moreover, the fabricated bimodal aerogels present the reduced shrinkage, density, and effective thermal conductivity of 6.3% and 0.063 g cm^−3^, 28.7 mW m^−1^ K^−1^, respectively. Furthermore, the bimodal polyimide/TPU aerogels show the higher porosity of 96.5 vol% along with increased mechanical flexibility over the conventional polyimide aerogel with comparable backbone chemistry.

## Abstract 2

Aerogels have presented a very high potential to be utilized as airborne nanoparticles' filtration media due to their nanoscale pore size and extremely high porosity.

## Graphical abstract

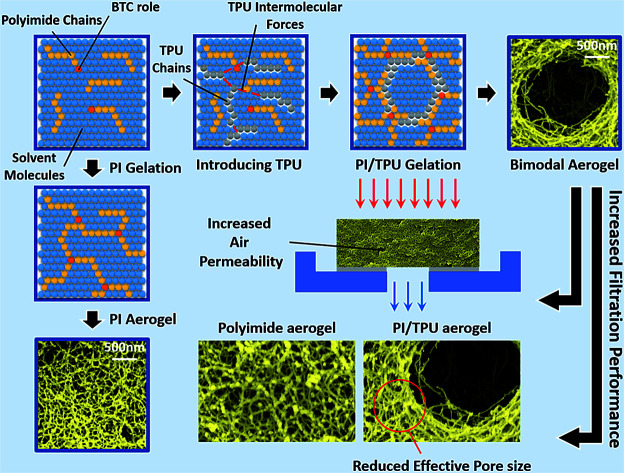

## Introduction

Fast penetration of modern technology in all aspects of human life along with reduced lifespan of the products, such as electronics, resulted in increasing the number of production sites all over the world and specifically in developing countries. This resulted in an excessive increase in air pollution in large industrial cities and became one of the main concerns in the world. The hazard level of airborne particles has an inverse relation with the particle size. Therefore, the smaller particles, specifically those with less than 300 nm diameter, are considered as the most dangerous particles for human health.^1^ Inhalation of such nanoscale particles may result in highly severe diseases such as lung cancer.^2^ This presents the importance of filtering such particles before spreading into the ecosystem and entering the human body. Concerning their nanoscale cell structure, high porosity of more than 80 vol%, and large surface area aerogels presented the great potential to be used as nanoscale airborne particle filtration materials.^3–7^

Aerogel can be formed from a large variety of chemicals by replacing the liquid content of a wet gel with air without damaging the gel solid network.^4,5,8,9^ Silica aerogels are the first and most widely studied type of aerogels.^10–14^ However, recently the organic polyimide aerogels presented higher potential to achieve enhanced mechanical properties and even flexibility over the previously studied aerogels.^9,15–17^ Moreover, the polyimide aerogels presented a higher service temperature of up to 400 °C over the other organic aerogels.^18–20^ Furthermore, moisture resistant polyimide aerogels are reported by using varying chemicals and specific monomers, such as dimethylbenzidine (DMBZ).^21–24^ These together suggested the high capability of polyimide aerogels to be used as airborne nanoparticle filters in different industrial applications with a wide range of environmental conditions and operating temperatures.^1,16,25^ Based on this, Jana *et al.*, is reported high filtration efficiency of 99.999% at the air permeability of 2.43 × 10^−10^ m^2^ from DMBZ–pyromellitic dianhydride (PMDA) polyimide aerogels with 10 wt% solid over solvent fraction.^1^

As discussed previously, aerogels' filtration performance such as permeability or filtration efficiency has a direct and sensitive relation with the aerogel nanostructure assembly including the cell size and morphology.^1,26,27^ Therefore, the fabrication of aerogel filters with the desired performance with respect to the industrial application requirements necessitates an accurate control of the aerogel nanostructure assembly formation. Reviewing the literature reveals that there were a very limited number of attempts in tailoring the nanostructure assembly of the polyimide aerogels, those were constrained to the change of the backbone chemistry, the crosslinking agent type, the solvent material, and the solid over the solvent weight fraction.^1,15,26,28^ However, due to the very limited control of such strategies on the gel-solid network configuration, such attempts were not effective in accurate tailoring of the aerogels nanostructure assembly and mainly resulted in the random alternation of the aerogel morphology. The aerogel nanostructure assembly is mainly formed during the initial gelation stage and with respect to the interaction of the involved molecules and the intermolecular forces in that stage.^15^ The formed solid skeleton of the gel will further develop and may experience some shrinkage during the next processing stages. However, its initial solid structural arrangement will mainly remain intact. This suggests that improving the filtration performance of the aerogels requires the control of the intermolecular forces during the initial gelation stage and as a result, tailoring the aerogels nanostructure configuration. However, with respect to the quantity and the number of involved parameters in molecular scale, analyzing and controlling of the sol–gel molecular interactions would be too complicated, and even cannot be modelled by currently available software.^29^

Given this background, in this work, the novel idea of tailoring the aerogel nanostructure assembly by disturbing the intermolecular forces during the initial gelation stage of sol–gel transition is implemented for the first time. This was achieved by introducing the thermoplastic polyurethane (TPU) polymer chains to the prepared polyimide oligomers solution. The proper fraction of TPU chains along with controlling the other parameters resulted in the formation of a uniform bimodal micro and nano porous aerogel structure. With respect to its nanostructure assembly, the fabricated bimodal aerogel can be used in airborne nanoparticles filtering and gas adsorption media applications with enhanced performance over the aerogel counterparts. Moreover, the fabricated polyimide/TPU aerogels presented reduced shrinkage, and density as well as increased porosity and mechanical flexibility over the polyimide aerogel with comparable backbone chemistry.

## Experimental

### Materials

In this study 4,4′-oxydianiline (ODA) and biphenyl-tetracarboxylic acid dianhydride (BPDA) are chosen as diamine and dianhydride monomers, respectively. Based on the previous works, the properties of the ODA–BPDA backbone polyimide aerogels are comprehensively studied.^15,23,26,30–32^ Therefore, with respect to its relatively higher elasticity, lower shrinkage, and fibrous morphology the ODA–BPDA backbone is selected in this work. *N*-Methylpyrrolidinone (NMP) is selected due to its high basic aprotic nature to improve the imidization reaction.^33–36^ Pyridine and acetic anhydride are utilized to catalyze the imidization and to scavenge water byproduct of the condensation reaction, respectively.^37–40^ 1,3,5-Benzenetricarbonyl tri-chloride (BTC) crosslinking agents is designated based on its ability to reduce the overall shrinkage along with enhancing the mechanical properties as well as the elasticity.^15,23^ All reagents are purchased from Sigma Aldrich and used without further purification.

### Characterization

A full parametric study is performed on the fabricated aerogels. The aerogel morphology is studied by field emission scanning electronic microscopy (Quanta, model FEG-250) operated at 5 and 15 kV. To achieve flat cross-sections of the samples, the samples are shear broken at liquid nitrogen. Then, the samples are sputter-coated with gold for the SEM analysis. The samples' pore size/distribution and specific surface area are determined by nitrogen adsorption–desorption isotherm analysis performed at 77 K, using an Autosorb iQ (Quantachrome Instruments) after degassing at 40 °C and 10^−3^ Pa for 24 h. Applying this analytical method, the adsorption and desorption isotherms of the N_2_ and the pore size and distribution are determined using the Density-Functional-Theory (DFT) and Barrett, Joyner, and Halenda (BJH) methods, as well as the samples' specific surface areas using the Brunauer–Emmett–Teller (BET) theory for 0.05 < *P*/*P*_0_ < 0.3. The air permeability of the fabricated aerogels is measured by the Frazier test using a vacuum pump and with respect to the pressure drop, volumetric flow rate, and the sample geometry.^1^ Porosity is measured by the Helium pycnometer (Quantachrome Instrument Ultra-Foam 1000) in accordance with the ASTM D6226 standard and at 15 psi. Density is measured using Denver Instrument electronic scale with the accuracy of 0.1 mg. The infrared spectroscopy is performed using a Platinum ATR spectrometer made by Bruker. Co. The thermo-gravimetric analysis is done using the thermo-gravimetric analyzer (TGA), TA-Instrument Q50, using the thermal ramp from room temperature to 750 °C and at the rate of 10 °C min^−1^ under the nitrogen environment. The effective thermal conductivity (*K*_eff_) is measured by Hot Disk (ThermTest Inc., TPS2500 S) under the ambient condition and at 23 °C, based on the Hot Disk Transient Plane Source method and according to the ISO/DIS 22007-2.2 standard. Instron 5848 micro-tester is employed to measure the mechanical compressive properties. The compression test is performed at the rate of 0.05 in min^−1^ and based on D695-02a ASTM guidelines. The Young's modulus is calculated based on the initial slope of the stress–strain curve.

### Samples preparation

**Fig. 1:**
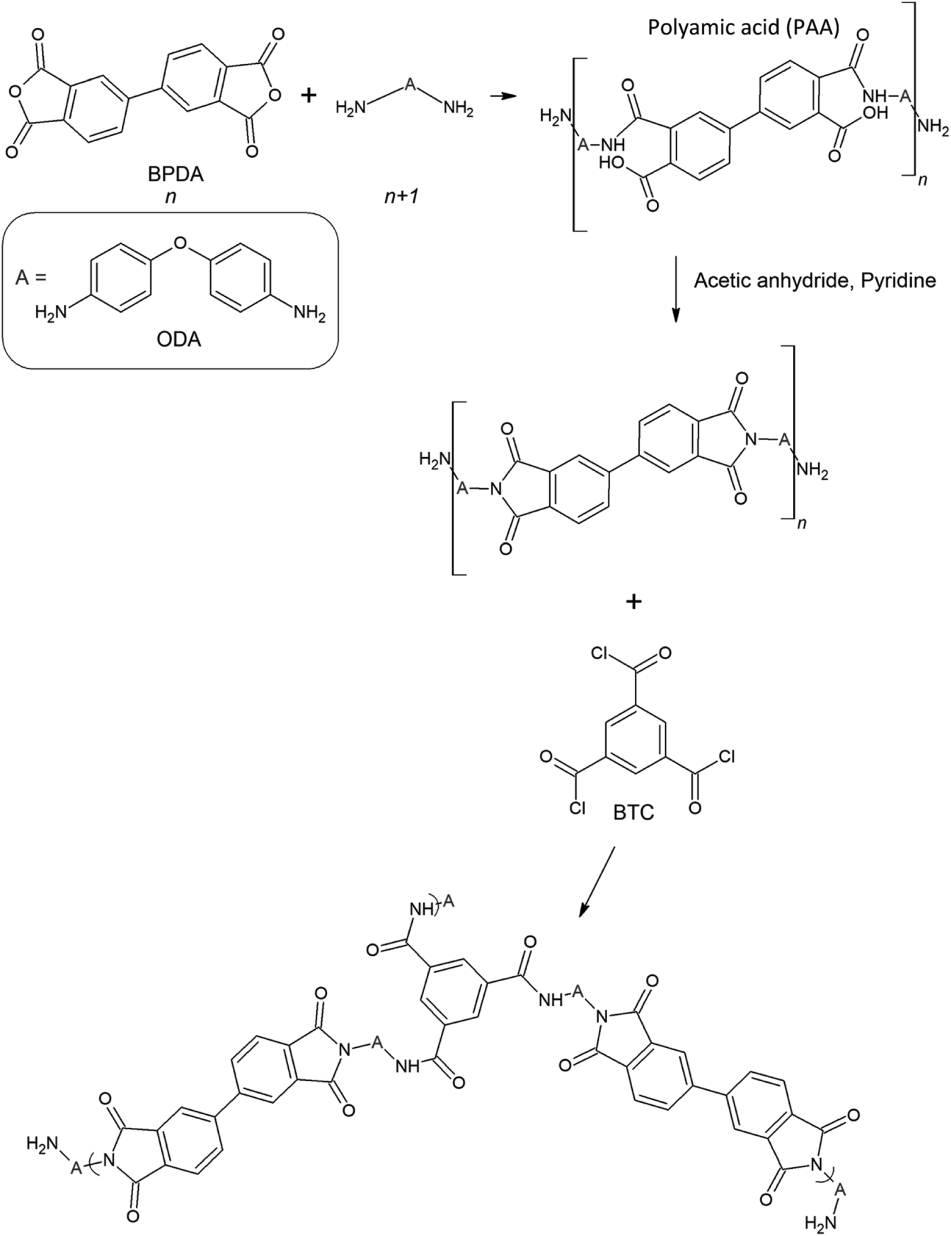
The schematic of the ODA–BPDA polyimide aerogels cross-linked by the BTC.^23^

With regards to the chemical role of the BTC in crosslinking of the amine end-capped polyimide oligomers, to achieve theoretically complete imidization without any unreacted monomers residue, the diamine over dianhydride molar ratio is calculated from eqn (1).1
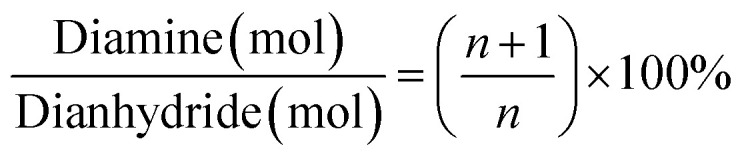
where “*n*” represents the number of repeat units in the oligomers. Moreover, to achieve optimum properties, the number of repeat units of *n* = 20 and solids over solvent weight fraction of 7% was selected from the previous works.^15,23,26,41^ As per previous works, the schematic of the imidization process and the BTC crosslinking role are presented in Fig. 1.^15,23^

To fabricate the gels, first all solids including the ODA, BPDA, BTC, and TPU content of the samples, if any, were separately dissolved in the NMP. After the solids were completely dissolved, then the two monomers' solutions were mixed and reaction for 5 minutes to form the polyamic acid (PAA) solution. This reaction was exothermic and increased the solution temperature. After that, the acetic anhydride and pyridine were added to the PAA solution and reacted for an additional 3 minutes. At this point, for the two sample-sets with the TPU content, the TPU solution was added and mixed with the polyimide solution for 1 minute. Finally, the BTC solution was added to the prepared solution followed by a further 3 minutes stirring, and before being poured into the prepared cylindrical syringe molds with 29 mm diameter and about 10 mm height.

After the gelation, the gels were kept in the mold for about 24 hours for ageing, and then the samples were carefully removed from the molds and placed in 100% NMP bath for another 24 hours to eliminate the acetic anhydride and pyridine residue. Due to the low solubility of CO_2_ in NMP, the NMP content of the samples is gradually exchanged with acetone in 24 hours intervals. After that, the samples were placed in a 100% acetone bath for a further 72 hours. Lastly, the prepared gels were dried by CO_2_ supercritical drying, using a 200 ml autoclave chamber. To perform the supercritical drying process, the gels were washed multiple times at 10 MPa pressure and 23 °C with liquid CO_2_ until the complete elimination of acetone. Finally, the chamber temperature was increased to 45 °C to convert the liquid CO_2_ into the supercritical state, and then the gaseous CO_2_ was vented out from the chamber at a very slow rate of 70 kPa min^−1^.

## Results and discussions

### Aerogel morphology, surface area, & pore size distribution

**Fig. 2:**
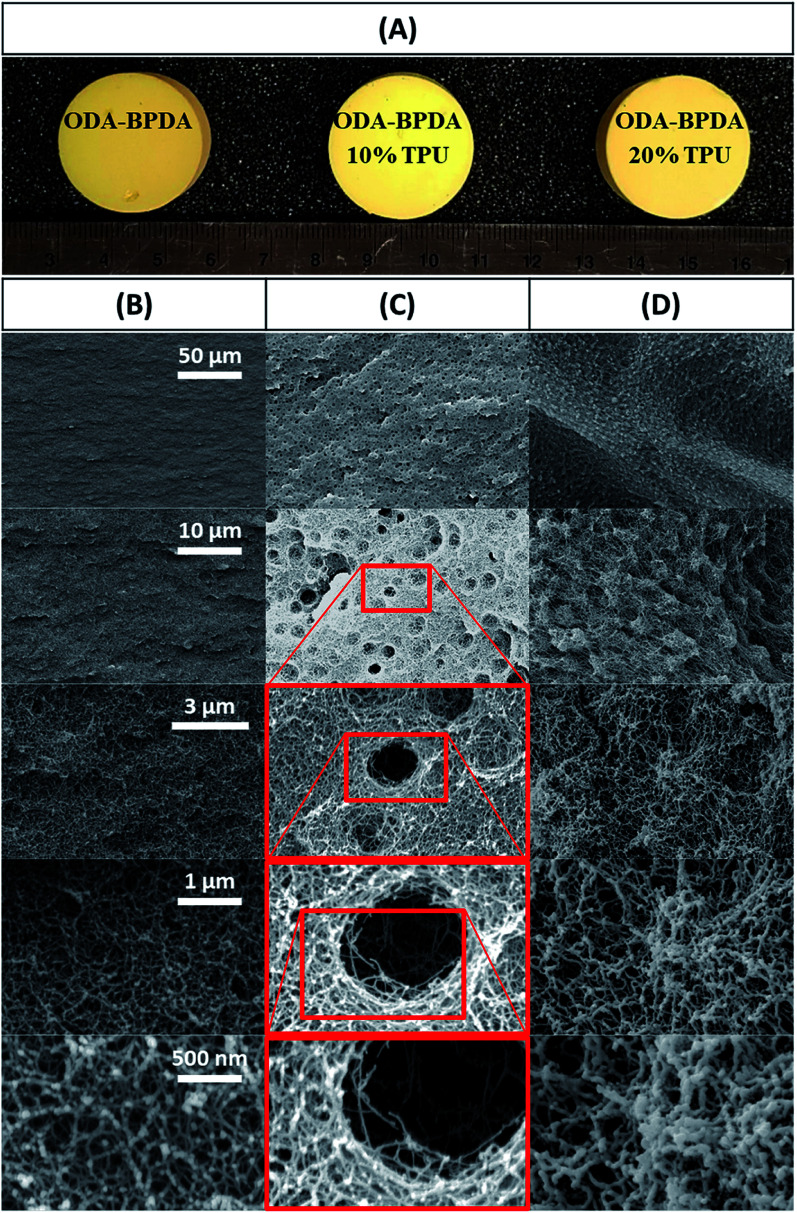
(A) picture of the fabricated aerogels, along with the SEM micrographs of (B) ODA–BPDA, (C) ODA–BPDA/10% TPU, & (D) ODA–BPDA/20% TPU samples.

As the properties of aerogel materials including the polyimides have very sensitive relations with their nanostructure assembly, controlling the aerogel's nanostructure configuration would be essential to improve the aerogel performance. However, the engagement of multiple complex parameters, such as the intermolecular interactions during the gelation, complicates the controlling of aerogel morphology. This poses a key challenge in improving the aerogels' performance. Though, it is not possible to fully analyze and control the aerogel nanostructure formation. In this work, polyimide aerogel morphology is tailored to a very uniform bimodal micro and nanopores structure by disturbing the intermolecular forces through a controlled process. This is achieved by introducing the optimum molar fraction of the TPU/NMP solution to the polyimide oligomers before the starting of the gelation stage. Fig. 2A presents the image of the fabricated ODA–BPDA aerogels with various TPU weight fractions. As presented in Fig. 2A, all fabricated aerogels represent a similar look with some variations in their yellow color intensity in macroscale. Fig. 2B–D present the scanning electron microscopy (SEM) micrograph of the fabricated aerogels at different magnifications, ranging from 1000 to 100 000 times. As observed from the SEM micrographs, varying TPU fraction resulted in different morphological configurations. The morphology of ODA–BPDA, ODA–BPDA/10 wt% TPU, and ODA–BPDA 20 wt% TPU samples are presented in Fig. 2B, C, and D, respectively. As, observed from Fig. 2B–D, all three sample sets presented very uniform morphologies at varying magnifications. However, the addition of the TPU chains resulted in altering the aerogel nanostructure assembly both in micro and nanoscale. As presented in Fig. 2B, the ODA–BPDA aerogels showed a homogeneous nanopores morphology with the average cell size of 40.00 ± 0.28 nm, as per the DFT analysis of the BET results. However, introducing the TPU solution to the ODA–BPDA polyimide chains resulted in the formation of a uniform micro size texture on the aerogel network structure. The addition of 10 wt% of TPU resulted in the formation of about 2.42 ± 0.39 μm diameter pores in ODA–BPDA/10% TPU samples. As presented in Fig. 2C, the formed micropores have a uniform distribution through the aerogel volume. Moreover, based on the Image-J software analysis, the micropores presented very similar pore diameters with a small standard deviation of 16%. From Fig. 2D, the further increase of the TPU content to 20 wt%, in ODA–BPDA/20% TPU samples, resulted in the aggregation of the TPU chains and formation of about 500 nm diameter knotted fibrous clusters. Altering the nanostructure assembly of the polyimide/TPU aerogels is achieved by the controlled disturbance of the intermolecular forces, such as van der Waals forces, of the polyimide chains by introducing the homogeneously dispersed TPU chains to the system at the right time and before the gelation stage. With respect to their intermolecular forces, the homogeneously dispersed TPU chains desire to agglomerate and to form TPU domains, while they are in solution state.^42^ Based on this phenomenon and the potential connections between the TPU and the polyimide chains, a controlled combination of the TPU and polyimide chains in the solution state is a successful strategy in tailoring the nanostructure assembly of the polyimide/TPU aerogels. The formation of TPU domains in ODA–BPDA/10% TPU and ODA–BPDA/20% TPU samples and as a result, lack of TPU in most aerogel volume is approved by the localized FTIR results to be discussed in the next sections. Moreover, this theory is fully in agreement with the homogenous dispersion of both micro voids and the knotted fiber clusters in ODA–BPDA/10% TPU and ODA–BPDA/20% TPU samples, respectively. With respect to this theory, the gelation time would be the other effective factor to control the morphology of the polyimide/TPU samples. Accelerating or delaying the gelation time either by changing the solution temperature or the solid over solvent fraction may alter the polyimide/TPU aerogels morphology by changing the provided time to incorporate the existing intermolecular forces.

The pore sizes, pore shapes, pore volume, pore classifications, and surface area are analyzed based on the N_2_ adsorption/desorption isotherms characterization and are summarized in Table 1.

**Table 1:** Summary of the BET, DFT, and BJH from the N_2_ adsorption/desorption analysis for the pore volume, the pore width, and the surface areas

Aerogel samples	*S*_BET_ (m^2^ g^−1^)	*S*_DFT_ (m^2^ g^−1^)	*V*_DFT_ (cc g^−1^)	*d*_DFT_ (nm)	*d*_BJH_ (nm)
**ODA–BPDA (neat)**	369.9	353.6	1.53 ± 0.28	40.00 ± 0.28	4.42
**ODA–BPDA/10% TPU**	481.2	376.0	2.60 ± 0.03	35.99 ± 0.03	3.51
**ODA–BPDA/20% TPU**	489.8	396.8	3.11 ± 0.04	35.98 ± 0.03	3.31

**Fig. 3:**
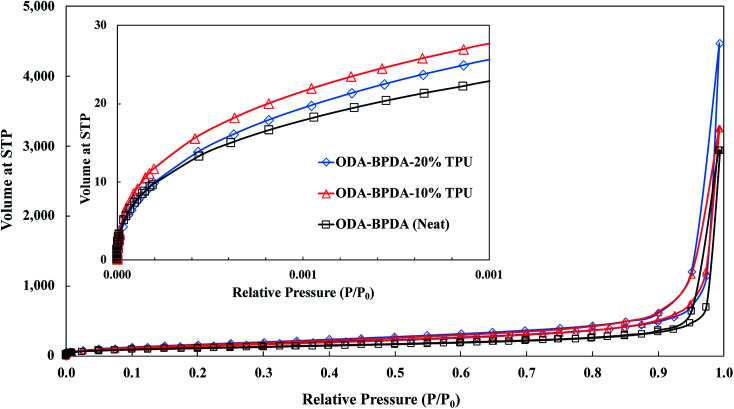
The nitrogen adsorption/desorption analysis for the ODA–BPDA (neat), ODA–BPDA–10% TPU, and ODA–BPDA–20% TPU samples.

**Fig. 4:**
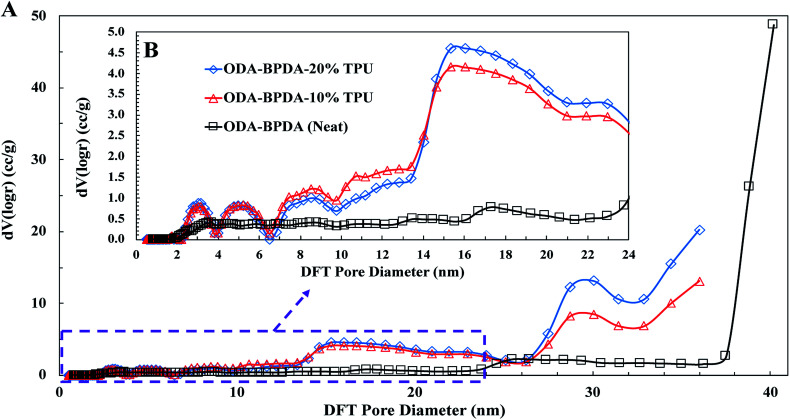
The DFT pore size distributions for the ODA–BPDA (neat), ODA–BPDA–10% TPU, and ODA–BPDA–20% TPU samples.

To further explore the nanoscale properties of fabricated aerogels it is essential to investigate the isotherm type, hysteresis loop, and its comparison with the IUPAC classification of pores. Thus the fabricated samples were further analyzed based on the comparison of the graph shapes obtained with the reference graphs on the IUPAC technical report on the gases' physisorption^43^. As presented in Fig. 3, all samples, with and without TPU, showed the H3 type hysteresis loop and type III isotherm shape. This type H3 hysteresis behavior is attributed to a relatively weak adsorbent–adsorbate interaction, which means that the molecular clustering is followed by pore filling at higher *P*/*P*_0_ where *P* represents equilibrium relative pressure and *P*_0_ is the saturation pressure against *P*. Fig. 4 shows the Density-Functional-Theory (DFT) results used to analyze the pore width and the pore size distributions of all the samples. Interestingly, the average pore size for neat samples (without TPU) was around 40 nm while the pore size for samples with TPU was around 36 nm. This shows that the addition of TPU independent of the amount, controlled the orientation of the chains affecting the pore size distribution. As the amount of TPU has increased from 10% to 20%, the surface area has slightly increased from 481 to 490 m^2^ g^−1^. This is evident that TPU chains were able to dictate the mesoporous characteristics leaving the final samples with dramatic differences in terms of morphology. As it was also evident in the SEM pictures, TPU chains tend to agglomerate due to their intermolecular forces in the solution state when the gelation has not been completed. The addition of TPU portion to the solution induced a higher tendency for the agglomeration, however the presence of polyimide chains and their interactions with TPU resulted in alteration of bimodal morphology (with 10% TPU) to knotted morphology (with 20% TPU).

Another interesting factor arises when comparing the pore filling behaviour of the samples under nitrogen. As shown in Fig. 3, isotherms show hysteresis, indicating the presence of significant mesopores while the mesopore size distributions (Fig. 4) were showing an extensive number of pores below 10 nm when TPU is present in samples, as shown in the inserted graph in Fig. 4. It can be also observed that the pore volume of those with width below 10 nm is much larger in samples having 10% TPU compared with neat and 20% TPU samples indicating the existence of bimodal characteristics within the samples. Besides, the pore size range of aerogel specimens produced with TPU was smaller. This trend shows that the addition of TPU leads to a much larger pore volume within the mesoporous samples. This indicates that the larger the TPU amount, the more open pores assessable for nitrogen absorption to be detected. This confirms that with the TPU presence and percentage the mesoporous morphology can be controlled to be whether knotted or bimodal.

### Shrinkage, density, and porosity

**Fig. 5:**
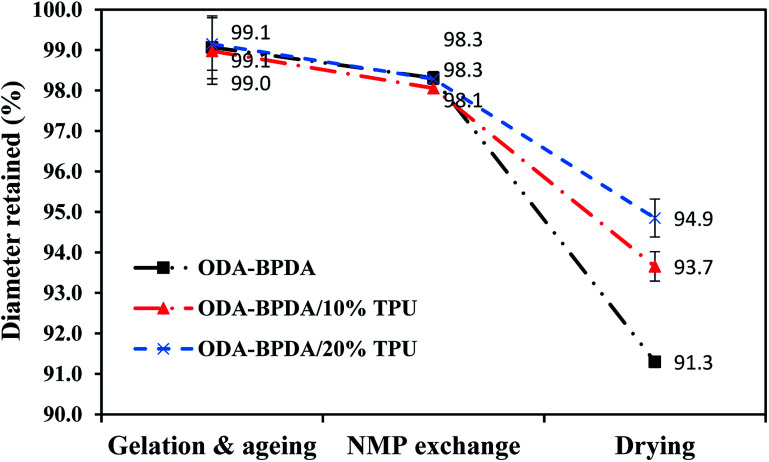
Retained diameter of ODA–BPDA aerogels with varying TPU weight fraction.

As aerogels owe many of their advantages to their high porosity, high shrinkage and as a result, reduced porosity would be a significant drawback in aerogel processing. Generally, it would be too complicated to fully explain the shrinkage behavior of the aerogels with respect to all affecting parameters. However, in simple terms the shrinkage behavior of the polyimide aerogels can be related to the three following factors: (i) the morphology of the gel–solid network including the crosslinks density and the chain length between the crosslinks, (ii) chains rigidities, and (iii) chains packing ability with respect to chain geometry and co-planarity.^15^ To study the shrinkage behavior of the fabricated aerogels during the processing, the retained diameter of the fabricated aerogels are measured at the three different processing stages of (i) after the 24 hours of ageing, (ii) after the completion of NMP to acetone solvent exchange, and (iii) after completion of the CO_2_ supercritical drying. Then, the results are compared with the initial sol–gel diameter, which is equal to the mold diameter. Fig. 5 presents the retained diameter trend of the fabricated aerogels at above mentioned processing stages.

As observed from Fig. 5, all the three samples with different TPU content presented very similar shrinkage either after aging or NMP to acetone solvent exchange. However, their shrinkage behavior was different after the elimination of the gel liquid content by the CO_2_ supercritical drying. Based on the previous studies, through the aerogel processing, samples usually experienced the highest shrinkage during the CO_2_ supercritical drying stage.^26^ The shrinkage during the drying stage is due to the elimination of the liquid content of the gel, which was assisting the cell's solid structure to resist against the shrinkage.^26^ As observed from Fig. 5, despite the aerogels' morphology, increasing TPU fraction resulted in reduced shrinkage. This clearly presented the role of the elastic TPU chains in reinforcing the polyimide chains to resist against the shrinkage after the elimination of the sample's liquid content.

Table 2 summarizes the properties of the fabricated ODA–BPDA aerogels with varying TPU content. As presented in Table 2, the addition of the TPU resulted in decreased density and increased porosity of the fabricated aerogels which agrees with the shrinkage results. However, the maximum porosity of 96.5% is observed from the ODA–BPDA/10 wt% TPU aerogels with bimodal pore structure. Reduced shrinkage and increased porosity of the aerogels with increasing TPU fraction is in agreement with the pore size distribution and surface area results of the samples.

**Table 2:** Properties of the studied polyimide aerogels with varying TPU fraction

	TPU (wt%)	Density (g cm^−3^)	Porosity (%)	Shrinkage (%)	Modulus (MPa)	Yield stress (MPa)	Thermal conductivity (mW mK^−1^)	Onset of decomposition (°C)
**ODA–BPDA**	0%	0.083	94.7 ± 0.13	8.7	3.00	0.29	32.5	583
**ODA–BPDA/10% TPU**	10%	0.063	96.5 ± 0.16	6.3	1.84	0.19	28.7	310
**ODA–BPDA/20% TPU**	20%	0.049	96 ± 0.13	5.1	1.00	0.06	29.9	310

### Chemical structures

**Fig. 6:**
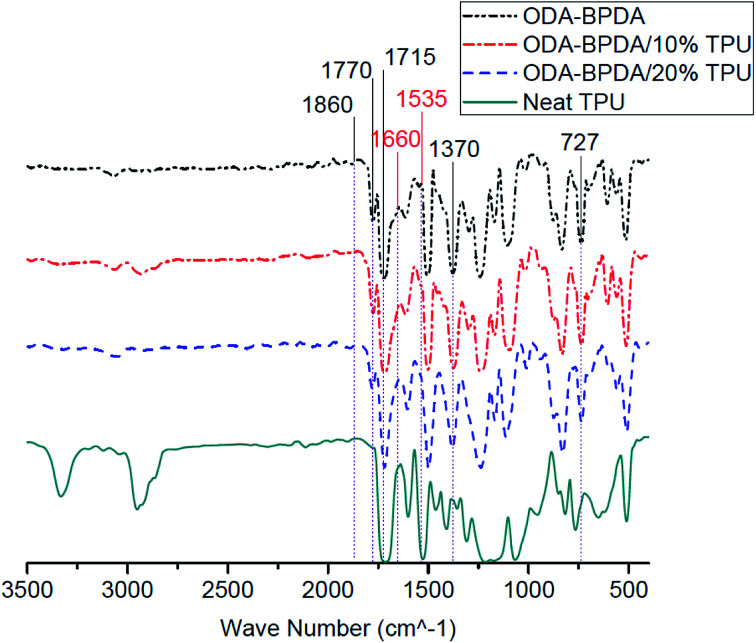
The Fourier transform infrared (FTIR) spectrum of fabricated aerogels along with the neat TPU.

Fig. 6 presents the Fourier transform infrared (FTIR) spectroscopy of the fabricated aerogels along with the neat TPU. All fabricated aerogels present similar characteristic bands of polyimides, including 727 cm^−1^, 1370 cm^−1^ (imide C–N), 1715 cm^−1^ (symmetric imide C=O) and 1770 cm^−1^ (asymmetric imide C=O) are observed for all studied samples.^44^ In addition, the lack of 1660 cm^−1^, 1535 cm^−1^, and of 1860 cm^−1^, bands on BPDA backbone aerogels may indicate the complete imidization of fabricated aerogels. As presented in Fig. 6, in most of the FTIR experiment trials, aerogels containing the TPU did not present the characteristic bands of the TPU and therefore, did not reveal any significant change in the FTIR bands compared to the neat ODA–BPDA aerogels. With respect to the confirmed TPU content of the samples by the TGA analysis, it can be obtained that the TPU is not homogenously dispersed in the structure. This shows that the TPU dispersion is controlled by the polymer chain interactions. Thus, TPU is located where the system energy was at the lowest. It is evident that TPU has a physical entanglement with the polyimide chains, inducing the special mesoporous structure. When the amount of TPU is increased from 10% to 20%, the physical entanglement is improved, and the mesoporous structure could be engineered from a bimodal structure to knitted structure. This is in agreement with the SEM micrographs.

### Thermal properties

**Fig. 7:**
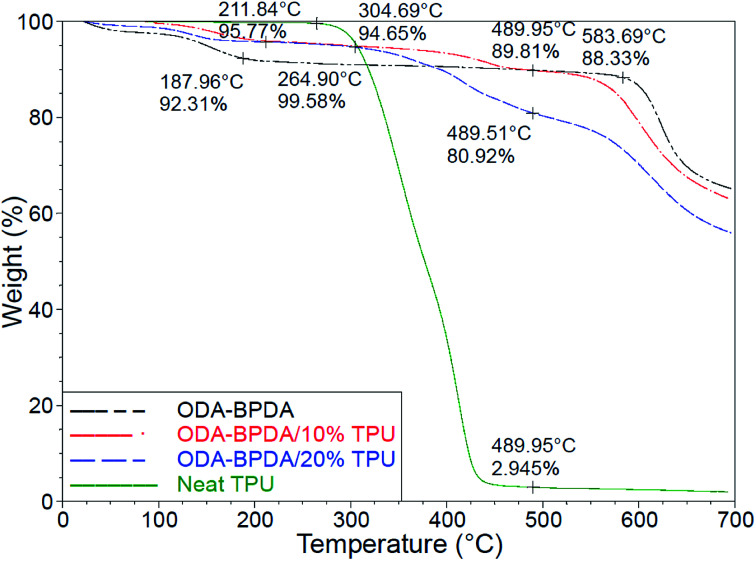
The TGA results fabricated aerogels and the neat TPU.

Fig. 7 presents the thermogravimetric analysis (TGA) of the fabricated aerogels along with the neat TPU. Among the organic materials, polyimides are well known for their high onset of decomposition and service temperature.^32^ However, the addition of the TPU with lower degradation temperature resulted in the reduced onset of decomposition of the fabricated aerogels. As observed from Fig. 7, the neat TPU and the ODA–BPDA aerogel presented the onset of decomposition of about 304 °C and 583 °C, respectively. The addition of the TPU to ODA–BPDA aerogel resulted in two slopes of decomposition on those samples. The neat ODA–BPDA aerogel presented the onset of decomposition 583 °C. The neat TPU presented the onset of decomposition at about 211 °C. The fabricated polyimide/TPU aerogels showed two decomposition trends, one started at about 304 °C and the second one started at about 575 °C, those are close to the TPU and ODA–BPDA aerogel decomposition temperatures, respectively. This clearly reveals that the samples contain a combination of TPU and ODA–BPDA chains. However, with respect to the weight loss trend at about 300 °C and between the first and the second decomposition slopes, the actual TPU content of the ODA–BPDA/10% TPU and ODA–BPDA/20% TPU samples can be estimated as 6.2 wt% and 15.5 wt%, respectively. Comparing this with the initially TPU weight fraction of the samples suggested that, about 22.5% to 38% of the dissolved TPU chains were probably washed away during the aerogel processing stages such as a solvent exchange. Furthermore, all three fabricated aerogels presented an initial weight loss ranging from 4 to 8% between 150 to 200 °C. A similar trend was observed from the previously studied polyimide aerogels and is believed to be due to the moisture content of the samples.^26^

With respect to their high porosity and mesoporous nanostructure assembly, aerogels may eliminate thermal conduction, convection, and even radiation.^45,46^ As a result, aerogels are well known with their low effective thermal conductivity (*K*_eff_) and super thermal insulation performance, with reported *K*_eff_ as low as 0.004 W m^−1^ K^−1^.^47^ From the previous works, polyimide aerogels have mainly presented the effective thermal conductivity ranging from 30 to 50 mW m^−1^ K^−1^.^48^ As presented in Table 2, the addition of the TPU to ODA–BPDA network resulted in reduced *K*_eff_ from 32.5 mW m^−1^ K^−1^ to about 29 mW m^−1^ K^−1^. Moreover, among the aerogels containing TPU, the bimodal ODA–BPDA/10% TPU samples presented lower *K*_eff_. This is believed to be due to the higher porosity and smaller pore size of the samples by the addition of the TPU. Reduced effective thermal conductivity with reduced pore size can be explained by the Knudsen effect and the comparable mean free path of the gas molecules with the aerogel cell size.^49^

### Mechanical properties

**Fig. 8:**
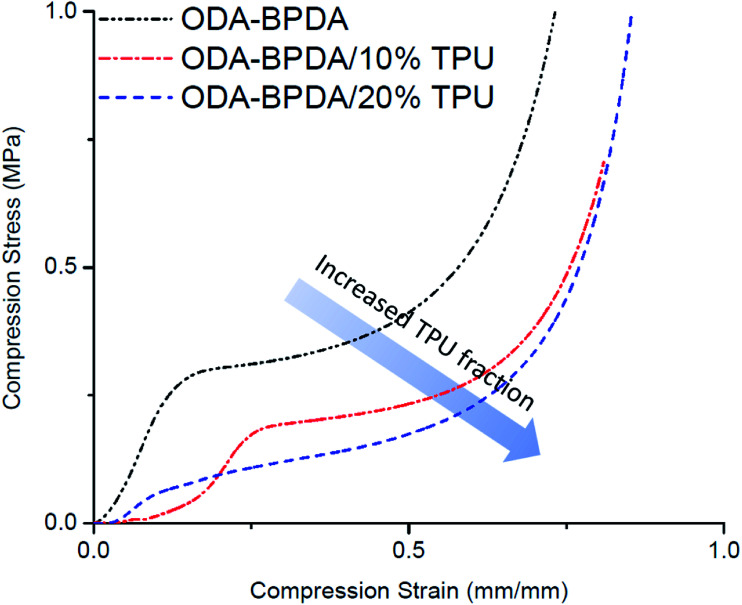
The stress–strain graph of the fabricated aerogels with varying TPU fraction.

To study the mechanical performance of the fabricated aerogels, the mechanical compression test is performed on the samples using the Instron micro-tester. The height of the samples was modified to about half of their diameter to avoid buckling. Fig. 8 presents the stress–strain behavior of the fabricated aerogels. Table 2 also represents the calculated yield stress and compression modulus of each fabricated aerogel sample. As presented in Fig. 8, increasing the TPU fraction of the ODA–BPDA polyimide aerogels resulted in reduced mechanical compression strength, modulus, and yield stress. This was partially due to the increased porosity of the fabricated polyimide/TPU aerogels compared to the neat polyimide aerogel. However, the reduced mechanical compression strength of the samples with increasing TPU content was also due to the increased fraction of the elastic TPU chains over the more rigid polyimide chains. In fact, the more elastic behavior of TPU chains resulted in lower modulus of the polyimide/TPU samples, as expected. The decrease on the yield strength of the aerogels when the amount of TPU is increased can also be attributed to the excessive physical entanglement of TPU chains through the main structure of the aerogel resulting into further local crack growth and air-cell closure and thus, faster yield of the material. It should be noted that the aerogels' compression modulus is calculated based on the initial slope of stress–strain curves and are presented in the Table 2.

### PPDA–PMDA aerogels network & air filtration application

**Fig. 9:**
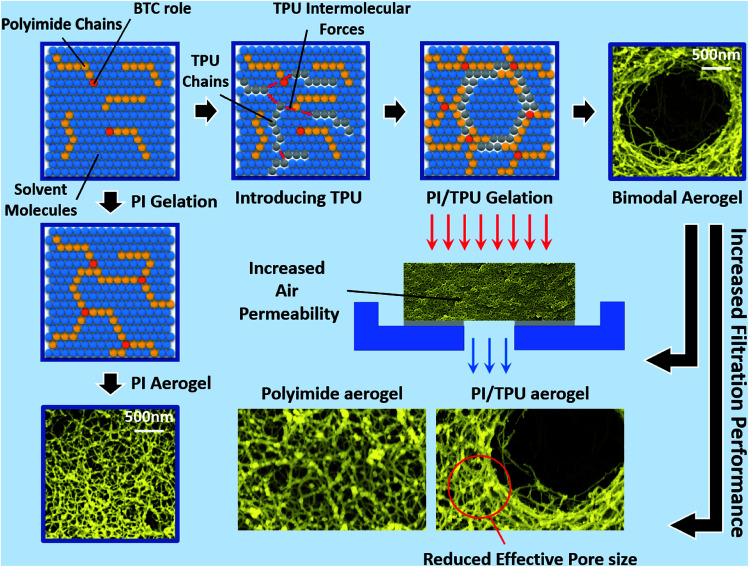
Bimodal polyimide/TPU aerogels with increased filtration performance over the typical polyimide aerogels.

The low shrinkage of 6.3%, high porosity of 96.5%, and the tailored bimodal micro and nanostructure assembly of the ODA–BPDA/20% TPU aerogels with reduced cell size suggest their potential to be used in airborne nanoparticles filtering applications. In this context, the air permeability of the fabricated aerogels is calculated based on the Frazier test theory using eqn (2).^1^2
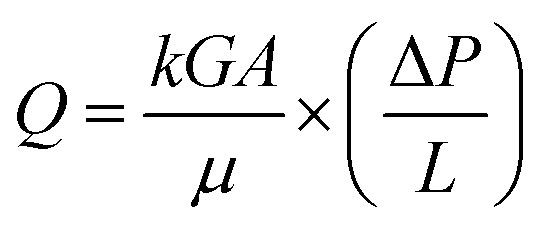
where *G*, *A*, and *L* represent the sample geometry and correspond to the sample shape factor, cross-sectional area of the sample normal to the air flow direction, and the sample thickness, respectively. Moreover, *Q* demonstrates to the volumetric air flow rate, Δ*P* represents the pressure change across the specimen, *k* corresponds to the permeability constant, and *μ* shows the viscosity of air.^1^ The term *μ* is calculated as 18.27 × 10^−6^ Pa s, with respect to the analyzing temperature of 23 °C. From the results, the air permeability ranging from 4.67 × 10^−10^ to 7.4 × 10^−10^ m^2^ is calculated for the samples. Among the fabricated aerogels the ODA–BPDA/10% TPU samples presented the highest air permeability of 7.4 × 10^−10^ m^2^, which shows more than 30% improvement over the neat ODA–BPDA aerogels with the air permeability of 5.67 × 10^−10^ m^2^. The improved air permeability of the ODA–BPDA/10% TPU samples is believed to be due to their bimodal micro and nano porous structure. Further increase of the TPU content in ODA–BPDA/20% TPU samples and as result, the knotted fiber clusters morphology resulted in reduced air permeability of 4.67 × 10^−10^ m^2^. Based on this, successful tailoring of the aerogel nanostructure assembly in ODA–BPDA/20% TPU samples resulted in 30% increased air permeability along with reduced average cell size over the neat ODA–BPDA aerogels. Therefore, the reduced effective pore size and increased air permeability suggest the improved filtration efficiency and filtration performance of the fabricated bimodal polyimide/TPU aerogels (Fig. 9).

## Conclusions

The unique physical, thermal, and mechanical properties of the aerogels presented a sensitive and direct relation with its nanostructure assembly and morphology. The nanostructure assembly of the aerogel is mainly formed during the gelation stage and with respect to the intermolecular forces in that stage. This suggests the complexity of tailoring aerogel nanostructure assembly and as a result, improving its performance. In this work, the nanostructure assembly of the ODA–BPDA aerogels is successfully tailored to a uniform bimodal micro and nano porous morphology. This was achieved by disturbance of the sol–gel intermolecular forces through a controlled process and by introducing the proper fraction of the homogeneously dispersed TPU chains to the ODA–BPDA chains in the solution state. Due to the intermolecular forces, such as van der Waals forces, the homogeneously dispersed TPU chains tend to agglomerate and to form TPU domains, while they are in the solution state. Based on this phenomenon and the originated connections between the TPU and the polyimide chains, controlled combining of the two systems was successful in tailoring the nanostructure assembly of the polyimide/TPU aerogels to a bimodal micro and nano porous morphology. The bimodal morphology of the fabricated aerogels, the TPU content of the samples, and congregation of the TPU chains along with formation of TPU domains were proved by SEM, TGA, and FTIR analysis, respectively. With respect to their bimodal micro and nano porous nanostructure assembly, the ODA–BPDA/10% TPU aerogels offered more than 30% improvement air permeability over the neat ODA–BPDA aerogels, while presented reduced pore size. This resulted in improved filtration performance of the fabricated ODA–BPDA/10% TPU aerogels over the previously studied polyimide aerogels. The presented strategy of tailoring aerogels nanostructure assembly by controlled disturbing of the sol–gel intermolecular forces is not limited to the studied polyimide aerogel and its application in this work. This strategy suggests an effective approach to be employed in tailoring the nanostructure assembly and as result, improving the properties of a wide range of aerogel materials with different industrial applications.

## Conflicts of interest

The authors declare that they have no known competing financial and non-financial interests that could have appeared to influence the work reported in this paper.

## Supplementary Material
